# Sexual Violence Associated With Sexual Identity and Gender Among California Adults Reporting Their Experiences as Adolescents and Young Adults

**DOI:** 10.1001/jamanetworkopen.2021.44266

**Published:** 2022-01-20

**Authors:** David J. Inwards-Breland, Nicole E. Johns, Anita Raj

**Affiliations:** 1 Rady Children’s Hospital, San Diego, California; 2 School of Medicine, University of California, La Jolla; 3 Center on Gender Equity and Health, Department of Medicine, University of California, La Jolla; 4 Division of Social Sciences, Department of Education Studies, University of California, La Jolla

## Abstract

**Importance:**

Disproportionate levels of violence exist in lesbian, gay, bisexual, queer, asexual, and other sexual identity (LGBQA+) communities, often starting in adolescence and young adulthood, but little research exists on the range of sexual violence (SV) experiences.

**Objective:**

To assess risk of experiences of sexual violence among California LGBQA+ adults during adolescence and young adulthood.

**Design, Setting, and Participants:**

This survey study analyzed data from Cal-VEX 2020, a state-representative survey of California residents aged 18 years and older, to assess associations between LGBQA+ identification and types of SV experienced in adolescence (ages 13-17 years) and young adulthood (ages 18-24 years) using gender-stratified adjusted logistic regression. Forms of SV included verbal, cyber, and coercion or physically aggressive sexual harassment; homophobic or transphobic slurs; and forced sex.

**Exposure:**

Sexual identity.

**Main Outcomes and Measures:**

The outcome of interest was experiences with any 5 forms of sexual violence (verbal sexual harassment, homophobic or transphobic slurs, cyber sexual harassment, sexual coercion or physically aggressive sexual harassment, and forced sex) during adolescence or young adulthood.

**Results:**

A total of 2102 contacted individuals (response rate, 26.2%) completed the Cal-VEX survey and were included in these analyses. Among these, the mean (SD) age was 46.6 (17.7) years, 1149 identified as female, 953 identified as male, and 214 respondents (9.6%) identified as LGBQA+. Compared with heterosexual individuals, LGBQA+ individuals had significantly higher odds of having experienced homophobic or transphobic slurs (women: AOR, 14.65; 95% CI, 5.14-41.77; men: AOR, 14.17; 95% CI, 6.96-28.86) and forced sex (women: AOR, 5.35; 95% CI, 2.74-10.43; men: AOR, 2.68; 95% CI, 1.01-7.10) in adolescence. LGBQA+ women also had higher odds of having experienced verbal and coercion or physically aggressive sexual harassment in adolescence (AOR, 2.33; 95% CI, 1.30-4.19). In young adulthood, LGBQA+ individuals also had higher odds of having experienced homophobic or transphobic slurs (women: AOR, 18.58; 95% CI, 7.12-48.49; men: AOR, 16.73; 95% CI, 8.26-33.92) in young adulthood. LGBQA+ men also had higher odds of having experienced homophobic or transphobic slurs (AOR, 16.73; 95% CI, 8.26-33.92); verbal (AOR, 3.29; 95% CI, 1.44-7.53), cyber (AOR, 6.32; 95% CI, 1.50-26.52), and coercion or physically aggressive (AOR, 5.54; 95% CI, 2.08-14) sexual harassment; and forced sex (AOR, 21.26; 95% CI, 5.63-80.35) in young adulthood.

**Conclusions and Relevance:**

This survey study found that adult LGBQA+ individuals were at increased risk for having experienced SV across the continuum during adolescence and young adulthood, consistent with prior research. Multifold solutions are needed to support LGBQA+ youth, including altering social norms accepting SV and homophobia, creating safer schools and other institutional environments, and supporting healthy sexual and romantic partnerships.

## Key Points

**Question:**

Are lesbian, gay, bisexual, queer, asexual, and other sexual identity (LGBQA+) youth at increased risk for experiencing violence across the sexual violence continuum?

**Findings:**

This survey study including 2102 participants found that adult LGBQA+ individuals were at increased risk for having experienced sexual violence across the continuum during adolescence and young adulthood.

**Meaning:**

These findings suggest the need for multifaceted solutions to support LGBQA+ youth, including altering social norms accepting sexual violence and homophobia, creating safer schools and other institutional environments, and supporting healthy sexual and romantic partnerships.

## Teaser

This survey study assesses sexual violence associated with sexual identity and gender among lesbian, gay, bisexual, queer, asexual, and other sexual identities (LGBQA+) adults reporting their experiences as adolescents and young adults.

## Introduction

Adolescents and young adults who are lesbian, gay, bisexual, questioning or queer, asexual, and other sexual identities (LGBQA+) have sexual orientations outside heterosexual norms and face discrimination and violence as a result. Nationally representative data from US high school students, from the Youth Risk Behavior Survey (YRBS) 2019, indicate that approximately 16% of adolescents identify as gay, lesbian, or bisexual or are unsure of their sexual identity.^1^ Because these data are the only representative data on the topic in the US, to our knowledge, they remain the standard regarding prevalence of gay, lesbian, bisexual, and unsure sexual identity among adolescence, but this is likely an underestimate. Among high school students who had no sexual contact, 86% identified as heterosexual and 9% identified as gay, lesbian, bisexual, or unsure.^1^ For these reasons, retrospective reporting on the experiences of LGBQA+ individuals in adolescence and young adulthood can be particularly insightful, particularly in terms of sensitive issues, such as experiences of sexual violence (SV), an issue disproportionately affecting LGBQA+ people.^2,3,4,5^

Stigmatization or abuse of those who do not adhere to the heterosexual norm remains all too common, affecting both those who identify as LGBQA+ and those who do not but act or are viewed outside of cisgender and heterosexual norms. Experiences of violence often begin in adolescence and young adulthood.^6,7^ Despite the resilience of LGBQA+ individuals and communities, these disproportionate levels of violence can create long-term negative physical and mental health consequences.^8,9^ Prevention of such violence is important for addressing recognized health disparities faced by LGBQA+ individuals^10^ and requires that we understand the scope and scale of violence experienced. National YRBS 2019 data show that adolescents who identify as gay, lesbian, bisexual, or unsure are more likely than adolescents who identify as heterosexual to have experienced SV in the past year (16%-20% vs 9%), with girls more likely than boys to report these abuses, except in the case of gay boys vs lesbian girls.^1^ While SV was defined broadly in this assessment, ie, “kissing, touching, or being physically forced to have sexual intercourse when they did not want to,”^1^ the use of a single item may result in underreporting, as well as inadequate clarity on LGBQA+ vulnerability, given likely underreporting or underrecognition of being LGBQA+ in this sample. Other research with adolescents has examined sexual identity and other forms of SV, including in-person and online sexual harassment, and again found greater risk for LGBQA+ youth, particularly boys, and this harassment is often inclusive of homophobic and transphobic name calling.^11,12^ Importantly, indications are that homophobic and transphobic harassment, viewed as sexual harassment including both sexual and gendered abuses,^13^ is the most common form of sexual harassment adolescent boys face in school.^14^ Notably, studies also show that perpetrators of homophobic and transphobic harassment in middle school are more likely to perpetrate sexual assault and rape in later adolescence,^15,16^ which again may disproportionately affect LGBQA+ youth, as indicated by YRBS data. None of these studies examined whether there were differences in observed associations by forms of SV. Furthermore, representative data on this topic with young adults remains largely lacking in the literature. These same associations may hold true in young adulthood but may be diminished in a context of greater autonomy and self-acceptance among young adults compared with adolescents.

Our study offers analysis of retrospective reports of SV in adolescence and young adulthood with data from a state-representative online survey of California adults, the California study on Violence Experiences Across the Lifespan 2020 study (Cal-VEX).^17^ To assess experiences of SV, we use the continuum of SV framework, which recognizes that sexual harm is not limited to the legal definitions of sexual offenses but rather include acts and contexts exerting unwanted sexual control or fear over an individual or group, with risk linked to gender and sexuality.^18^ Hence, our analysis assesses a range of sexual abuses, including verbal sexual harassment, homophobic or transphobic slurs, cyber sexual harassment, physically aggressive or coercive sexual harassment, and forced sex, by gender and sexual identity. More specifically, we hypothesize that LGBQA+ individuals, compared with heterosexual individuals, are more likely to report SV across the continuum in adolescence and young adulthood, and observed outcomes may be stronger for men even though women are more likely to experience SV. Additionally, we hypothesize that observed associations may be stronger in adolescence compared with young adulthood, given the more restrictive contexts of adolescence in both family and school environments, although associations would exist in both life stages.

## Methods

All research procedures for this survey study were approved by both NORC at the University of Chicago and the University of California San Diego institutional review board. Participants provided written informed consent for initial panel participation, and the NORC institutional review board approved a waiver of documentation of consent for this specific survey participation, as is standard for NORC surveys. All data were deidentified by NORC prior to provision of the data for analysis. The NORC team contacted participants to invite them into the online survey, which took approximately 15 minutes to complete. Participants were able to decline questions or stop the survey at any time. Given the sensitivity of the survey items, all participants were provided with information regarding victim services and a hotline number. This study is reported following the American Association for Public Opinion Research (AAPOR) reporting guideline.

This survey study uses data from the Cal-VEX Study, a state-representative online survey on violence experiences conducted with California adults aged 18 years and older, in March 2020.^17^ The Cal-VEX survey was administered by the survey research firm NORC at the University of Chicago^19^ using their online probability panel (AmeriSpeak), which uses an address-based sampling approach to recruit participants and supplemented this sample with additional nonprobability opt-in panels to reach the desired sample size.

NORC performed statistical calibration to combine these probability and nonprobability samples and created a survey-weighted final sample that is representative of the California adult population with regards to several key sociodemographic factors. We confirmed the representativeness of the weighted sample via comparison to published census and other state government data on gender, race and ethnicity, education, employment, income, age, sexual identity, citizenship, and disability status; all examined characteristics confirmed state-representativeness. Further detail on sampling, data calibration, and weighting methods is published elsewhere,^17^ and additional detail can be found in the eAppendix in the Supplement. The weighted AAPOR RR3 recruitment rate for this study was 24%, the weighted household retention rate was 85.6%, and the response rate to this survey was 26.2%. These are standard for online panel surveys, which typically range from 20% to 25%.^20^ Ultimately, the weighted AAPOR RR3 cumulative response rate was 5.4% (eAppendix in the Supplement).

Our primary outcomes of interest were experiences of 5 forms of SV in adolescence (defined as age 13-17 years) and young adulthood (defined as age 18-24 years). The forms of SV were verbal sexual harassment, homophobic or transphobic slurs, cyber sexual harassment, sexual coercion or physically aggressive sexual harassment, and forced sex. Definitions for each form of violence can be found in Table 1; the full definitions were provided as prompts within the survey. Respondents were asked whether they had ever experienced each form of violence; if they answered yes, they were then asked whether they had experienced it in childhood (age 0-12 years), adolescence (age 13-17 years), young adulthood (age 18-24 years), and/or adulthood (age ≥25 years). Our team created this measure based on the continuum of SV framework^18^ and prior research and theory on sexual harassment.^13^ This measure has been validated and used in prior publications.^13,14,21,22^

**Table 1.** Definitions of Types of Sexual Violence and Corresponding Experiences Provided in Survey

Type	Definition
Verbal sexual harassment	Include someone whistling, leering or staring at you, or calling out to you in ways that make you feel disrespected or unsafe. It can include someone talking about your body parts (such as your butt or breasts) inappropriately or offensively or saying sexually explicit comments or questions (“I want to do BLANK to you”). It can also include someone repeatedly asking you for a date or your phone number when you’ve said No.
Homophobic or transphobic slurs	Include someone misgendering you or calling you a homophobic or transphobic slur, like “Fag,” “Dyke,” or “Tranny.”
Cyber sexual harassment	Include someone electronically sending you or showing you sexual content without your permission, such as over e-mail, snapchat or Facebook or on their phone or computer. This can also include someone taking and/or sharing sexual pictures or videos of you without your permission.
Coercion or physically aggressive sexual harassment	Coercion can include someone forcing or pressuring you to do a sexual act in exchange for something (such as a good grade, a promotion, a job, drugs, food, money, or something similar) or instead of something (like paying rent or a citation, etc.).
	Physically aggressive sexual harassment can include someone flashing or exposing their genitals to you without your permission. This can also include someone purposely touching you or brushing up against you in an unwelcome, sexual way.
Forced sex	Include someone forcing you to do a sexual act without your permission or one that you don’t want to do (including while you are under the influence of alcohol or drugs).

Our independent variable was sexual identity. We asked: “Which of the following best represents how you think of yourself? Lesbian or gay; Straight, that is, not lesbian or gay; Bisexual; Something else.” We dichotomized responses to heterosexual or LGBQA+ sexual identity. Only 1 participant declined to respond and was dropped from analysis.

Our stratification variable was self-reported gender, assessed as “How do you describe yourself? Male; female; transgender; do not identify as male, female, or transgender.” Owing to small numbers and to ensure anonymity, 5 individuals who indicated transgender identity and 7 individuals who indicated other gender identity were excluded from these analyses. Demographic covariates included current age (categorized as 18-29, 30-44, 45-59, and ≥60 years), race and ethnicity (categorized as Asian, Black, Latinx, White, or other or multiple races [including American Indian or Alaska Native, Native Hawaiian or Pacific Islander, and those who answered “some other race”]), and income (dichotomized as lowest income quintile or not). Race and ethnicity were included in analyses given disparities in violence experiences across racial and ethnic identities, as well as known intersectional risks associated with sexual identity, gender, and race and ethnicity. Other or multiple race included 9 American Indian or Alaska Native respondents, 13 Native Hawaiian or Pacific Islander respondents, and 28 individuals who selected the answer choice “some other race” (no further detail provided); 72 respondents selected multiple races.

### Statistical Analysis

We present frequencies (survey-weighted percentages and unweighted counts) of included demographic characteristics and experiences of SV in adolescence and young adulthood for the total sample, by gender and by sexual identity. We used Pearson χ^2^ tests to assess bivariate differences in demographic characteristics and experiences of violence by gender and by sexual identity. We then conducted a series of adjusted logistic regression analyses stratified by gender to assess the association of sexual identity with each of 5 forms of SV, first in adolescence and then in young adulthood, including age, race and ethnicity, and income. As an additional post hoc analysis, we limited the sample to respondents aged 25 years and older (as those aged 18-24 years had less time at risk for the young adult experiences) and replicated unadjusted comparisons and adjusted regression analyses. All regression analyses accounted for survey design and weighting to produce state-representative findings and were conducted using Stata statistical software version 15.1 (StataCorp). *P* values were 2-sided, and statistical significance was set at *P* < .05. Analyses were initiated in December 2020 and finalized in April 2021.

## Results

### Characteristics of the Sample

A total of 2102 individuals participated in the survey; 13 participants were removed from the final analytic sample because of missing data. For our weighted analytic sample, 48.1% of participants identified as men (953 participants unweighted) and 51.9% identified as women (1139 participants unweighted, and 9.6% identified as LGBQA+ (214 participants unweighted) (Table 2). A total of 207 participants (12.9%) were Asian, 135 participants (5.6%) were Black, 450 participants (32.8%) were Latinx, and 1188 participants (42.1%) were White. By income quintile, 409 participants (21.6%) were in the lowest quintile, indicating they were living in poverty. LGBQA+ identity was significantly associated with age and race and ethnicity: LGBQA+ individuals were more likely than heterosexual individuals to be aged 18 to 44 and to be multiracial or other race (Table 2).

**Table 2.** Sample Characteristics by Gender and Sexual Identity

Characteristic	No. (%)^a^						
	Overall (N = 2102)	Gender			Sexual identity		
		Female (n = 1149)	Male (n = 953)	*P* value^b^	Heterosexual (n = 1888)	LGBQA+ (n = 214)	*P* value^c^
Gender							
Female	1149 (51.9)	NA	NA	NA	1043 (52.4)	106 (46.7)	.26
Male	953 (48.1)	NA	NA		845 (47.6)	108 (53.3)	
Sexual identity							
Heterosexual	1888 (90.4)	1043 (91.4)	845 (89.4)	.26	NA	NA	NA
LGBQA+	214 (9.6)	106 (8.6)	108 (10.6)		NA	NA	
Age, y							
18-29	438 (21.3)	263 (23.6)	175 (18.7)	.24	377 (20.7)	61 (26.6)	.02
30-44	610 (28.6)	331 (28.1)	279 (29.1)		543 (27.7)	67 (36.9)	
45-59	456 (24.2)	252 (23.8)	204 (24.7)		410 (24.7)	46 (19.3)	
≥60	598 (26.0)	303 (24.6)	295 (27.5)		558 (26.9)	40 (17.3)	
Race and ethnicity							
Asian	207 (12.9)	108 (12.6)	99 (13.2)	.41	197 (13.4)	10 (8.7)	.02
Black	135 (5.6)	72 (4.4)	63 (6.9)		125 (5.8)	10 (3.8)	
Latinx	450 (32.8)	270 (33.6)	180 (31.9)		393 (32.4)	57 (36.9)	
White	1188 (42.1)	626 (42.3)	562 (41.8)		1073 (42.7)	115 (36.3)	
Other or multiple races^d^	122 (6.6)	73 (7.1)	49 (6.1)		100 (5.8)	22 (14.4)	
Lowest income quintile							
No	1693 (78.4)	894 (75.3)	799 (81.8)	.02	1533 (79.2)	160 (71.0)	.07
Yes	409 (21.6)	255 (24.7)	154 (18.2)		355 (20.8)	54 (29.0)	

Abbreviations: LGBQA+, lesbian, gay, bisexual, queer, asexual, and other sexual identity; NA, not applicable.

^a^ Numbers are unweighted, percentages are weighted to be representative of the California population.

^b^ χ^2^ test of female vs male.

^c^ χ^2^ test of heterosexual vs LGBQA+.

^d^ Other race and ethnicity includes American Indian, Alaska Native, Pacific Islander, or other open-text response.

### Experiences of SV in Adolescence and Young Adulthood

The most common form of SV reported in both adolescence and young adulthood was verbal sexual harassment, reported by 370 participants (16.4%) during adolescence and 422 participants (16.6%) during young adulthood (Table 3). Prevalence of forms of SV did not differ significantly between adolescence and young adulthood. There was a small but not significant increase in sexual coercion or physically aggressive sexual harassment with age, reported by 186 participants (7.2%) in adolescence and 219 participants (9.2%) in young adulthood (*P* = .07).

**Table 3.** Experiences of Sexual Violence in Adolescence and Young Adulthood Overall, by Gender, and by Sexual Identity

Experience	No. (%)^a^							
	Overall (N = 2102)	Gender			Sexual identity			
		Female (n = 1149)	Male (n = 953)	*P* value^b^	Heterosexual (n = 1888)	LGBQA+ (n = 214)	*P* value^c^
**Adolescence (age 13-17 y)**							
Verbal sexual harassment	370 (16.4)	317 (26.3)	53 (5.8)	<.001	310 (15.7)	60 (23.7)	.01
Homophobic/transphobic slurs	107 (5.2)	28 (2.3)	79 (8.4)	<.001	49 (2.8)	58 (28.1)	<.001
Cyber sexual harassment	78 (4.7)	55 (5.5)	23 (4.0)	.34	59 (4.5)	19 (7.2)	.12
Coercion or physically aggressive sexual harassment	186 (7.2)	149 (11.3)	37 (2.8)	<.001	153 (6.6)	33 (12.7)	.01
Forced sex	87 (3.3)	76 (5.3)	11 (1.1)	<.001	60 (2.3)	27 (12.0)	<.001
**Young adult (age 18-24 y)**							
Verbal sexual harassment	422 (16.6)	366 (27.4)	56 (5.1)	<.001	354 (15.7)	68 (25.0)	.005
Homophobic/transphobic slurs	100 (4.5)	37 (3.0)	63 (6.2)	.01	42 (2.2)	58 (26.2)	<.001
Cyber sexual harassment	70 (3.4)	48 (3.9)	22 (2.7)	.28	53 (2.9)	17 (8.1)	.004
Coercion or physically aggressive sexual harassment	219 (9.2)	183 (12.7)	36 (5.5)	<.001	176 (8.2)	43 (18.4)	<.001
Forced sex	129 (4.6)	113 (7.5)	16 (1.4)	<.001	102 (3.9)	27 (10.7)	<.001

Abbreviation: LGBQA+, lesbian, gay, bisexual, queer, asexual, and other sexual identity.

^a^ Numbers are unweighted, percentages are weighted to be representative of the California population.

^b^ χ^2^ test of female vs male.

^c^ χ^2^ test of heterosexual vs LGBQA+.

Experience of homophobic or transphobic slurs was significantly more likely among men, whereas all other forms of sexual harassment, coercion, and violence were significantly more likely among women, reported in both adolescence and young adulthood (Table 3). All assessed forms of sexual harassment and violence, except for cyber sexual harassment, were significantly more frequently reported by those who identified as LGBQA+ compared with heterosexual individuals. Similar findings were seen in experiences of violence in young adulthood, except cyber sexual harassment was also significantly more likely for LGBQA+ individuals compared with heterosexual participants.

### Adjusted Regression Models to Assess Associations Between LGBQA+ Identities and Experiences of SV in Adolescence

Adjusted analyses, accounting for age, race and ethnicity, and income, indicate that among women, LGBQA+ individuals had higher odds of having experienced verbal sexual harassment (AOR, 1.74; 95% CI, 1.02-2.97), homophobic or transphobic slurs (AOR, 14.65; 95% CI, 5.14-41.77), sexual coercion or physically aggressive harassment (AOR, 2.33; 95% CI, 1.30-4.19), and forced sex (AOR, 5.35; 95% CI, 2.74-10.43) in adolescence (Table 4). Among men, LGBQA+ participants had higher odds of having experienced homophobic or transphobic slurs (AOR, 14.17; 95% CI, 6.96-28.86) and forced sex (AOR, 2.68; 95% CI, 1.01-7.10).

**Table 4.** Adjusted Logistic Regression Models to Assess Associations Between Sexual Identity and Experiences of Sexual Violence in Adolescence Adjusting for Demographic Covariates Among California Adults

Characteristic	Verbal sexual harassment				Homophobic slurs				Cyber sexual harassment				Coercion or physically aggressive sexual harassment				Forced sex			
	Female		Male		Female		Male		Female		Male		Female		Male		Female		Male	
	AOR (95% CI)	*P* value	AOR (95% CI)	*P* value	AOR (95% CI)	*P* value	AOR (95% CI)	*P* value	AOR (95% CI)	*P* value	AOR (95% CI)	*P* value	AOR (95% CI)	*P* value	AOR (95% CI)	*P* value	AOR (95% CI)	*P* value	AOR (95% CI)	*P* value
Sexual identity																				
Heterosexual	1 [Reference]	.04	1 [Reference]	.16	1 [Reference]	<.001	1 [Reference]	<.001	1 [Reference]	.51	1 [Reference]	.71	1 [Reference]	.005	1 [Reference]	.048	1 [Reference]	<.001	1 [Reference]	.01
LGBQA+	1.74 (1.02-2.97)		1.84 (0.78-4.36)		14.65 (5.14-41.77)		14.17 (6.96-28.86)		1.35 (0.55-3.29)		1.26 (0.37-4.22)		2.33 (1.30-4.19)		2.68 (1.01-7.10)		5.35 (2.74-10.43)		9.42 (1.85-47.91)	
Age, y																				
18-29	1 [Reference]	NA	1 [Reference]	NA	1 [Reference]	NA	1 [Reference]	NA	1 [Reference]	NA	1 [Reference]	NA	1 [Reference]	NA	1 [Reference]	NA	1 [Reference]	NA	1 [Reference]	NA
30-44	0.47 (0.29-0.78)	.003	0.99 (0.40-2.46)	.98	0.13 (0.03-0.46)	.002	0.77 (0.27-2.20)	.62	0.19 (0.08-0.47)	<.001	0.28 (0.08-1.00)	.049	1.13 (0.58-2.18]	.72	0.45 (0.09-2.20]	.33	0.43 (0.18-1.06]	.07	3.03 (0.49-18.64]	.23
45-59	0.55 (0.32-0.95)	.03	0.65 (0.16-2.69)	.55	0.23 (0.07-0.80)	.02	0.70 (0.25-2.02)	.51	NA	NA	0.32 (0.06-1.67)	.18	1.01 (0.54-1.88)	.98	0.48 (0.11-2.08)	.33	1.46 (0.59-3.63)	.42	NA	NA
≥60	0.30 (0.17-0.52)	<.001	0.17 (0.05-0.58)	.005	0.08 (0.01-0.49)	.01	0.52 (0.18-1.50)	.23	NA	NA	NA		1.10 (0.53-2.29)	.80	0.39 (0.09-1.78)	.22	0.85 (0.35-2.06)	.73	0.70 (0.07-6.50)	.75
Race and ethnicity																				
Asian	0.29 (0.15-0.58)	<.001	1.60 (0.60-4.30)	.35	0.11 (0.01-0.94)	.04	1.93 (0.61-6.10)	.26	0.41 (0.09-1.82)	.24	5.30 (1.06-26.61)	.04	0.29 (0.11-0.76)	.01	1.27 (0.25-6.40)	.77	NA		NA	NA
Black	0.42 (0.19-0.95)	.04	1.86 (0.25-13.63)	.54	0.59 (0.06-6.12)	.66	NA	NA	0.09 (0.01-0.76)	.03	9.42 (0.93-95.08)	.06	0.55 (0.18-1.70)	.30	0.35 (0.07-1.74)	.20	0.21 (0.05-0.81)	.02	0.33 (0.03-3.35)	.35
Latinx	0.71 (0.45-1.13)	.15	0.73 (0.24-2.19)	.57	0.77 (0.21-2.89)	.70	0.87 (0.43-1.78)	.71	0.37 (0.14-0.95)	.04	2.96 (0.59-14.79)	.19	0.68 (0.35-1.31)	.25	0.46 (0.18-1.16)	.10	0.63 (0.29-1.36)	.24	1.22 (0.24-6.24)	.81
White	1 [Reference]	NA	1 [Reference]	NA	1 [Reference]	NA	1 [Reference]	NA	1 [Reference]	NA	1 [Reference]	NA	1 [Reference]	NA	1 [Reference]	NA	1 [Reference]	NA	1 [Reference]	NA
Other and multiple races^a^	0.64 (0.33-1.23)	.18	0.96 (0.29-3.24)	.95	1.20 (0.30-4.81)	.79	0.72 (0.13-4.02)	.70	0.42 (0.12-1.46)	.17	0.85 (0.08-9.11)	.89	0.93 (0.42-2.05)	.86	0.83 (0.18-3.82)	.81	0.73 (0.22-2.40)	.60	NA	NA
Lowest income quintile																				
No	1 [Reference]	.11	1 [Reference]	.60	1 [Reference]	.04	1 [Reference]	.62	1 [Reference]	.58	1 [Reference]	.64	1 [Reference]	.17	1 [Reference]	.64	1 [Reference]	.95	1 [Reference]	.09
Yes	0.68 (0.42-1.10)		1.34 (0.45-4.04)		0.23 (0.06-0.94)		1.25 (0.51-3.09)		0.77 (0.30-1.95)		1.32 (0.41-4.20)		0.68 (0.39-1.18)		1.38 (0.36-5.22)		0.98 (0.42-2.26)		4.26 (0.81-22.51)	

Abbreviations: AOR, adjusted odds ratio; LGBQA+, lesbian, gay, bisexual, queer, or other sexual identity; NA, not applicable.

^a^ Other race and ethnicity includes American Indian, Alaska Native, Pacific Islander, or other open-text response.

We found that among women, some racial and ethnic groups experienced lower risk for SV in adolescence compared with White individuals (eg, cyber sexual harassment among Black women: AOR, 0.09; 95% CI, 0.01-0.76). However, among men, there was increased risk for SV in adolescence among some racial and ethnic groups compared with White individuals (eg, cyber sexual harassment among Asian men: AOR, 5.30; 95% CI, 1.06-26.61). Among women, there was a negative association between having low income and having experienced homophobic or transphobic slurs in adolescence (AOR, 0.23; 95% CI, 0.06-0.94).

### Adjusted Regression Models to Assess Associations Between Sexual Identity and Experiences of Violence in Young Adulthood

Adjusted analyses, accounting for age, race and ethnicity, and income, indicated that among women, LGBQA+ individuals had higher odds of having experienced homophobic or transphobic slurs in young adulthood (AOR, 18.58; 95% CI, 7.12-48.49) (Table 5). Among men, LGBQA+ individuals had higher odds of having experienced all forms of violence in young adulthood, including verbal sexual harassment (AOR, 3.29; 95% CI, 1.44-7.53), homophobic or transphobic slurs (AOR, 16.73; 95% CI, 8.26-33.92), cyber sexual harassment (AOR, 6.32; 95%, 1.50-26.52), sexual coercion or physically aggressive sexual harassment (AOR, 5.54; 95% CI, 2.08-14.76), and forced sex (AOR, 21.26; 95% CI, 5.63-80.35).

**Table 5.** Adjusted Logistic Regression Models to Assess Associations Between Sexual Identity and Experiences of Sexual Violence in Young Adulthood Adjusting for Demographic Covariates Among California Adults

Characteristic	Verbal sexual harassment				Homophobic slurs				Cyber sexual harassment				Coercion or physically aggressive sexual harassment				Forced sex			
	Female		Male		Female		Male		Female		Male		Female		Male		Female		Male	
	AOR (95% CI)	*P* value	AOR (95% CI)	*P* value	AOR (95% CI)	*P* value	AOR (95% CI)	*P* value	AOR (95% CI)	*P* value	AOR (95% CI)	*P* value	AOR (95% CI)	*P* value	AOR (95% CI)	*P* value	AOR (95% CI)	*P* value	AOR (95% CI)	*P* value
Sexual identity																				
Heterosexual	1 [Reference]	.06	1 [Reference]	.005	1 [Reference]	<.001	1 [Reference]	<.001	1 [Reference]	.57	1 [Reference]	.01	1 [Reference]	.18	1 [Reference]	.001	1 [Reference]		1 [Reference]	<.001
LGBQA+	1.66 (0.99-2.79)		3.29 (1.44-7.53)		18.58 (7.12-48.49)		16.73 (8.26-33.92)		1.30 (0.52-3.24)		6.32 (1.50-26.52)		1.52 (0.83-2.80)		5.54 (2.08-14.76)		1.65 (0.81-3.37)	.17	21.26 (5.63-80.35)	
Age																				
18-29	1 [Reference]	NA	1 [Reference]	NA	1 [Reference]	NA	1 [Reference]	NA	1 [Reference]	NA	1 [Reference]	NA	1 [Reference]	NA	1 [Reference]	NA	1 [Reference]	NA	1 [Reference]	NA
30-44	0.73 (0.46-1.17)	.20	0.67 (0.28-1.57)	.35	0.14 (0.03-0.55)	.005	0.78 (0.24-2.53)	.67	0.50 (0.21-1.18)	.11	0.54 (0.17-1.70)	.29	1.21 (0.66-2.21)	.54	0.72 (0.19-2.77)	.64	1.02 (0.47-2.20)	.96	0.49 (0.10-2.36)	.37
45-59	0.60 (0.35-1.03)	.07	0.30 (0.11-0.88)	.03	0.97 (0.31-3.04)	.96	1.74 (0.53-5.64)	.36	0.02 (0.00-0.16)	<.001	0.02 (0.00-0.21)	.001	1.38 (0.72-2.64)	.33	1.13 (0.20-6.40)	.89	1.01 (0.45-2.28)	.98	0.71 (0.06-7.95)	.78
≥60	0.55 (0.31-0.97)	.04	0.08 (0.02-0.25)	<.001	0.10 (0.02-0.45)	.002	0.92 (0.26-3.25)	.90	0.01 (0.00-0.06)	<.001	0.19 (0.05-0.73)	.02	0.81 (0.43-1.51)	.50	0.88 (0.20-3.80)	.86	0.77 (0.37-1.62)	.49	0.67 (0.09-4.97)	.69
Race and ethnicity																				
Asian	0.38 (0.21-0.67)	.001	1.50 (0.49-4.56)	.48	2.19 (0.44-10.95)	.34	1.37 (0.36-5.19)	.64	0.35 (0.10-1.22)	.10	4.09 (0.74-22.45)	.11	0.43 (0.18-1.01)	.052	3.18 (0.80-12.56)	.10	0.16 (0.04-0.70)	.02	NA	NA
Black	0.57 (0.28-1.13)	.11	0.30 (0.05-1.68)	.17	0.26 (0.03-2.54)	.25	NA	NA	0.10 (0.01-0.90)	.04	7.80 (1.31-46.45)	.02	0.65 (0.25-1.68)	.37	6.06 (1.41-26.01)	.02	1.36 (0.37-4.94)	.64	2.73 (0.34-22.22)	.35
Latinx	0.51 (0.32-0.81)	.005	0.78 (0.34-1.75)	.54	0.51 (0.15-1.76)	.29	0.71 (0.29-1.73)	.45	0.50 (0.19-1.27)	.14	1.19 (0.33-4.25)	.79	0.71 (0.42-1.19)	.19	3.34 (1.15-9.74)	.03	0.40 (0.20-0.79)	.01	1.99 (0.39-10.10)	.41
White	1 [Reference]	NA	1 [Reference]	NA	1 [Reference]	NA	1 [Reference]	NA	1 [Reference]	NA	1 [Reference]	NA	1 [Reference]	NA	1 [Reference]	NA	1 [Reference]	NA	1 [Reference]	NA
Other or multiple races^a^	1.06 (0.57-2.00)	.85	1.25 (0.32-4.94)	.75	0.49 (0.08-3.02)	.44	0.20 (0.04-1.09)	.06	0.46 (0.12-1.74)	.25	0.68 (0.06-7.19)	.75	2.01 (0.98-4.10)	.06	1.16 (0.19-7.00)	.97	1.17 (0.50-2.74)	.71	1.16 (0.11-11.73)	.90
Low income (lowest quintile)																				
No	1 [Reference]	.03	1 [Reference]	.52	1 [Reference]	.54	1 [Reference]	.007	1 [Reference]	.74	1 [Reference]	.75	1 [Reference]	.51	1 [Reference]	.10	1 [Reference]	.37	1 [Reference]	.23
Yes	0.6 (0.38-0.95)		1.36 (0.53-3.47)		1.49 (0.42-5.30)		3.70 (1.43-9.60)		0.86 (0.36-2.06)		0.77 (0.16-3.72)		0.83 (0.48-1.44)		2.78 (0.81-9.56)		1.35 (0.70-2.58)		2.12 (0.62-7.22)	

Abbreviations: AOR, adjusted odds ratio; LGBQA+, lesbian, gay, bisexual, queer, or other sexual identity; NA, not applicable.

^a^ Other race and ethnicity includes American Indian, Alaska Native, Pacific Islander, or other open-text response.

We found that among women, some racial and ethnic groups had lower risk for having experienced verbal sexual harassment and forced sex in young adulthood compared with White individuals (eg, verbal sexual harassment among Asian women: AOR, 0.38; 95% CI, 0.21-0.67). Among men, there was an increased risk for having experienced SV in young adulthood among some racial and ethnic groups compared with White men (eg, coercion or physically aggressive sexual harassment among Black men: AOR, 6.06; 95% CI, 1.41-26.01). Among women, there was a negative association between having low income and having experienced verbal sexual harassment in young adulthood (AOR, 0.60; 95% CI, 0.38-0.95). Among men, there was a positive association between having low income and having experienced homophobic or transphobic slurs in young adulthood (AOR, 3.70; 95% CI, 1.43-9.60).

### Analyses Limited to Age 25 Years and Older

The magnitude and significance of unadjusted and adjusted findings reported above remained mostly consistent when 241 respondents ages 18 to 24 were removed, with the exception of results for verbal sexual harassment. In the age-restricted sample, there were no longer significantly different experiences of verbal sexual harassment in adolescence (262 heterosexual participants [14.7%] vs 48 LGBQA+ participants [20.1%]; *P* = .09) or young adulthood (315 heterosexual participants [15.5%] vs 54 LGBQA+ participants [21.3%]; *P* = .08) in unadjusted comparisons. In adjusted models, only the findings for women’s experiences in adolescence changed meaningfully (AOR, 1.68; 95% CI 0.92-3.11; *P* = .10).

## Discussion

In this survey study using a state-representative sample of California adults, we documented increased risk of having experienced several forms of SV among LGBQA+ individuals. Findings from our study among this representative sample of adults in California were generally consistent with previous research showing LGBQA+ populations were at greater risk for SV in adolescence and young adulthood than their heterosexual counterparts.^1,2,3,4,5^ However, this work extends prior research by documenting a broad range of SV experiences, as well as by documenting intersectional risks related to sexual identity and gender, and how these change from adolescence to young adulthood. Prior work has documented the importance of examining the intersection of gender with other inequalities and oppressions (eg, sexuality, gender identity, ethnicity, indigeneity, immigration status, disability) in terms of risk for violence.^23^ Our work demonstrates the association between being LGBQA+ and having experienced SV in adolescence was stronger for women than men, but this association was stronger for men in young adulthood. Further, in terms of SV in young adulthood, associations among men were specific to more severe forms of SV, such as coercive or physically aggressive sexual harassment and forced sex. This is consistent with national data from adults indicating increased risk for SV for gay and bisexual men.^5^

These intersectional gender differences are not what we hypothesized and may be related to shifts in sexual and romantic partnerships from adolescence to young adulthood. SV in adulthood is more likely to occur at the hands of a partner than a stranger or family member, and men are more likely than women to be perpetrators of SV.^17^ For LGBQA+ individuals, opposite-sex partners or delayed same-sex partnerships may be more likely in adolescence than young adulthood, which suggests that LGBQA+ individuals may feel more comfortable identifying as LGBQA+ and having same-sex partnerships in young adulthood than in adolescence. Hence, it may be through an increase in male partnership for LGBQA+ men and a decrease in male partnership for LGBQA+ women that risks shift in the gender by sexual identity interaction. These findings support prior research recommending shifts in male gender norms to reduce violence,^24,25^ and suggest such an approach may be meaningful for LGBQA+ as well as heterosexual men.

An additional important issue is the level and risk for homophobic and transphobic remarks LGBQA+ individuals face in adolescence and young adulthood, a time where their sexual identity and sense of value for self are forming. Approximately 1 in 20 people in the adult California population as a whole have experienced such offensive remarks in adolescence and young adulthood, with men more likely than women to report this. Among LGBQA+ individuals in our study, more than 1 in 4 individuals reported such abuse in adolescence and in young adulthood. These findings reinforce prior research documenting the pervasiveness of homophobic and transphobic harassment that persists among young people, especially boys.^14^ Recognition of such behavior as hate speech and ensuring there is not tolerance or support of it in institutional settings, such as schools, is a first step toward combatting this problem. Such behaviors contribute to worse educational outcomes, including missed school days and school dropout for LGBTQ+ adolescents compared with cisgender and heterosexual adolescents.^26^ Research suggests respectful and inclusive LGBTQ+ policies and gay-straight alliances in schools are associated with reducing these types of offenses, improving educational and well-being outcomes for youth, and supporting a positive school climate.^27^

Our findings also demonstrated race and ethnicity by gender and income by gender associations with our outcomes of interest. Among men, we found that being Asian, Black, or Latinx, compared with being White, was associated with higher odds of having experienced cyber sexual harassment, and being in the lowest income quintile relative to the highest income quintile was associated with higher odds of having experienced forced sex. These findings are consistent with prior research in terms of Black and Latinx men, compared with White men, being at greater risk for experiencing SV, although not specific to cyber sexual harassment.^28,29^ Differences by race and ethnicity and sexual identity have also been observed, in which LGBQA+ youths of Black or Asian backgrounds are less likely than White or Latinx LGBQA+ youths to experienced victimization from peers.^30,31^ Our findings also correspond with research demonstrating that violence is higher in resource-constrained communities of color, such as Black men who have sex with men.^32^ However, we did not find elevated risks associated with race and ethnicity among women; in fact, we found lower risk for some outcomes. These findings speak to the intersectional nature of social factors and risk for SV and the need for further research on whether intersections of racial, ethnic, and income disparities with sexual identity are associated with SV. We anticipate these findings would be salient, given prior work demonstrating the associations of race, ethnicity, and income interacting with sexual identity associations with health outcomes, including depression, anxiety, substance abuse, and HIV acquisition, as well as increased cancer risk.^6,7,9,28,29,30,32,33,34,35^

### Limitations

While our study findings offer important insight into the issues of SV for LGBQA+ individuals, they should be considered in terms of several limitations. Our data relied on self-report and therefore were subject to recall and social desirability biases. It is likely that SV was underreported, particularly among men and racial and ethnic minority groups.^36^ Our survey sample only included adults and asked them about their past experiences, which may not represent situations currently experienced by adolescents or young adults. We were limited in our assessments of the age ranges 13 to 17 years and 18 to 24 years owing to survey item setup, although other age ranges may be more theoretically or clinically meaningful. We had an insufficient number of respondents to fully explore differences by specific sexual identities, or by gender identity beyond male/female or by specific race and ethnicity, areas requiring further research. Thus, larger-scale survey research, as well as smaller-scale research focused on LGBQA+ and transgender individuals, is needed to understand these issues with greater reflection on the variation across identities. Additionally, while our response rate of 26% was comparable to other online surveys, the low response rate reduces the representativeness and generalizability of our findings, as does the participation in an online survey panel. For example, individuals who are institutionalized or experiencing homelessness and individuals who do not speak English or Spanish are not represented by this data. Along these lines, the wide 95% CIs seen in our male subsample are likely due to inadequate power owing to lower reports of these forms of SV among men.

## Conclusions

The findings of this survey study are consistent with prior research demonstrating LGBQA+ individuals were at increased risk for having experienced violence in adolescence and young adulthood and expand this work by documenting these risks across the continuum of SV and by demonstrating important gender by sexual identity intersectional differences. These differences include stronger associations with SV for girls in adolescence and for men in young adulthood. We also found that men of color and men in the lowest income quintile faced greater risks for having experienced SV compared with White men and men with higher income, indicating how social marginalization based on identity, race and ethnicity, and income are associated with men’s risk for SV. Findings also speak to the pervasiveness of homophobic slurs, especially for boys and men, which is not simply disrespectful but can be harmful for identity development and self-worth.

Violence occurs on a continuum of beliefs and actions, and the bases of this continuum are individual beliefs and social norms that allow SV to occur, and more so for socially vulnerable populations, such as LGBQA+ youth. While we recognize heterosexual-identified individuals can hold these norms rigidly, so too can LGBQA+ youth, and in the process be even more vulnerable to SV, including sexual discriminatory offenses. These findings speak to the need for multifold solutions to support LGBQA+ adolescents and young adults, including altering social norms accepting SV and homophobia, creating safer school and other institutional environments for LGBQA+ youth, and supporting healthy sexual and romantic partnerships for LGBQA+ people.

## Notes

Supplement. **eAppendix.** Harassment Survey 2020—CA: University Of California–San Diego: AmeriSpeak Field Report
