# Supplementary material for: Sexual Violence Associated With Sexual Identity and Gender Among California Adults Reporting Their Experiences as Adolescents and Young Adults
Source: JAMA Netw Open. 2022 Jan 20;5(1):e2144266. doi: 10.1001/jamanetworkopen.2021.44266 (PMC8777565; doi:10.1001/jamanetworkopen.2021.44266)
Supplement: Supplement. — eAppendix. Harassment Survey 2020—CA: University Of California–San Diego: AmeriSpeak Field Report [file jamanetwopen-e2144266-s001.pdf]

Supplemental Online Content

Inwards-Breland DJ, Johns NE, Raj A. Sexual violence associated with sexual identity and gender among California adults reporting their experiences as adolescents and young adults. *JAMA Netw Open*. 2022;5(1):e2144266.
doi:10.1001/jamanetworkopen.2021.44266

eAppendix. Harassment Survey 2020—CA: University Of California–San Diego: Amerispeak Field Report

This supplemental material has been provided by the authors to give readers additional information about their work.

© 2022 Inwards-Breland DJ et al. *JAMA Network Open*.

HARASSMENT SURVEY 2020 - CA
UNIVERSITY OF CALIFORNIA – SAN DIEGO

AMERISPEAK FIELD REPORT

April 24, 2020

Client Contact: Anita Raj
NORC Account Manager:
Bruce Barr | Barr-Bruce@norc.org
NORC Project Manager:
LeeAnn McCabe | McCabe-LeeAnn@norc.org

© 2022 Inwards-Breland DJ et al. *JAMA Network Open*.

STUDY INTRODUCTION

NORC conducted the Harassment Survey 2020-CA on behalf of the University of California – San Diego using NORC's AmeriSpeak® Panel for the sample source. Supplemental sample was obtained from two non-probability sample sources, Dynata and Lucid. This research was done to support research about violence, abuse and harassment in early 2020 in the State of California. Subject matter will focus on abuse & harassment in general, not just sexual.

This study was offered in English and Spanish and was conducted online only.

For more detailed information on the AmeriSpeak panel recruitment and management methodology, please see the Appendix ("Technical Notes on AmeriSpeak Methodology") attached to this AmeriSpeak Field Report.

STUDY-SPECIFIC DETAILS

Sampling

A general population sample of California adults age 18 and older was selected from NORC's AmeriSpeak Panel for this study.

The sample for a specific study is selected from the AmeriSpeak Panel using sampling strata based on age, race/Hispanic ethnicity, education, and gender (48 sampling strata in total). The size of the selected sample per sampling stratum is determined by the population distribution for each stratum. In addition, sample selection takes into account expected differential survey completion rates by demographic groups so that the set of panel members with a completed interview for a study is a representative sample of the target population. If panel household has one more than one active adult panel member, only one adult in the household is eligible for selection (random within-household sampling). Panelists selected for an AmeriSpeak study earlier in the business week are not eligible for sample selection until the following business week.

For technical information about the AmeriSpeak Panel, including recruitment process and panel management policies, please see the Appendix.

The AmeriSpeak panel sample was supplemented with respondents from the Dynata and Lucid nonprobability online opt-in panels.

Field

A small sample of English-speaking Lucid panelists were invited on March 6 for a pretest. In total, NORC collected 68 pretest interviews. The initial data from the pretest was reviewed by NORC and a delivered to Anita Raj.

No changes were made before fielding the Main survey to collect the 2,115 interviews.

Response Rate Reporting for AmeriSpeak sample only

Weighted AAPOR RR3 Recruitment rate: 24.1%

Weighted Household retention rate: 85.6%

Survey completion rate: 26.2%

Weighted AAPOR RR3 cumulative response rate: 5.4%

© 2022 Inwards-Breland DJ et al. *JAMA Network Open*.

Gaining Cooperation of AmeriSpeak Panelists for the Study

To encourage study cooperation, NORC sent email reminders to sampled web-mode panelists on the following dates:

- Monday, March 23

Panelists were offered the cash equivalent of $2 for completing this survey.

Data processing

NORC prepared a fully labeled data file of respondent survey data and demographic data for Anita Raj.

NORC applied cleaning rules to the survey data for quality control. Respondents that finished the survey in less than a third of the median duration and/or skipped over fifty percent of the questions shown to them were removed from the data set.

Weighting

NORC calculated panel weights for the completed AmeriSpeak Panel and nonprobability online interviews, as described below. First we describe the calculation of the weights for the AmeriSpeak sample, and then describe the statistical corrections made to the non-probability sample via NORC's TrueNorth™ calibration weighting service.

AmeriSpeak Sample

Generally speaking, the steps for calculating the weights for the AmeriSpeak Panel interviews involves the following sequential steps: incorporating the appropriate probability of selection, and then incorporating nonresponse and raking ratio adjustments (to population benchmarks).

For the AmeriSpeak Panel interviews, study-specific base weights are derived from the final panel weight and the probability of selection from the panel under the study sample design. Since not all sampled panel members responded to the interview, an adjustment is needed to compensate for survey non-respondents. This adjustment decreases potential nonresponse bias associated with sampled panel members who did not respond to the interview for the study. A weighting class approach is used to adjust the weights for survey respondents to represent non-respondents.

At this stage of weighting, any extreme weights were trimmed using a power transformation to minimize the mean squared error, and then, weights were re-raked to the same population totals.

TrueNorth Calibration for Nonprobability Sample

In order to incorporate the nonprobability sample, NORC used TrueNorth calibration services, an innovative hybrid calibration approach developed at NORC based on small area estimation methods in order to explicitly account for potential bias associated with the nonprobability sample[1,2]. The purpose of TrueNorth calibration is to adjust the weights for the nonprobability sample so as to bring weighted distributions of the

---

[1] Ganesh, N., Pineau, V., Chakraborty, A., Dennis, J.M., (2017). "Combining Probability and Non-Probability Samples Using Small Area Estimation." *Joint Statistical Meetings 2017 Proceedings.*

[2] Yang, Y. Michael, Nada Ganesh, Ed Mulrow, and Vicki Pineau. (2018). "Estimation Methods for Nonprobability Samples with a Companion Probability Sample," Proceedings of the Joint Statistical Meetings, 2018.

© 2022 Inwards-Breland DJ et al. *JAMA Network Open*.

nonprobability sample in line with the population distribution for characteristics correlated with the survey variables. Such calibration adjustments help to reduce potential bias, yielding more accurate population estimates.

The weighted AmeriSpeak sample and the calibrated nonprobability sample were used to develop a small area model to support domain-level estimates, where the domains were defined by race/ethnicity, age, and gender. The dependent variables for the models were key survey variables. The model included covariates, domain-level random effects, and sampling errors. The covariates were external data available from other national surveys such as health insurance, internet access, voting behavior, and housing type from the American Community Survey (ACS) or the Current Population Survey (CPS).

Finally, the combined AmeriSpeak and nonprobability sample weights were derived such that for the combined sample, the weighted estimate reproduced the small domain estimates (derived using the small area model) for key survey variables.

Design Effect and Sampling Margin of Error Calculations

Study design effect: 1.96468

Study margin of error: +/- 2.99%

Under TrueNorth, the margins of error were estimated from the root mean squared error associated with the small area model, along with other statistical adjustments. A TrueNorth estimate of margin of error is a measure of uncertainty that accounts for the variability associated with the probability sample as well as the potential bias associated with the nonprobability sample.

Deliverables

The following files were created for Anita Raj as part of the study deliverables:

- Survey interview data file in both SAS and SPSS formats
- Codebook in Excel format
- Final programming questionnaire in Word document
- Field report documenting study procedures
- One set of banner tables in Excel formats

HOW TO DESCRIBE AMERISPEAK AND NORC @ THE UNIVERSITY OF CHICAGO

For purposes of publication, when describing AmeriSpeak and its panel methodology, we recommend using the following language:

> Funded and operated by NORC at the University of Chicago, **AmeriSpeak®** is a probability-based panel designed to be representative of the US household population. Randomly selected US households are sampled using area probability and address-based sampling, with a known, non-zero probability of selection from the NORC National Sample Frame. These sampled households are then contacted by US mail, telephone, and field interviewers (face to face). The panel provides sample coverage of approximately 97% of the U.S. household population. Those excluded from the sample include people with P.O. Box only addresses, some addresses not listed in the USPS Delivery Sequence File, and some newly constructed dwellings. While most AmeriSpeak households participate in surveys by web, non-internet households can participate in AmeriSpeak

© 2022 Inwards-Breland DJ et al. *JAMA Network Open*.

surveys by telephone. Households without conventional internet access but having web access via smartphones are allowed to participate in AmeriSpeak surveys by web. AmeriSpeak panelists participate in NORC studies or studies conducted by NORC on behalf of governmental agencies, academic researchers, and media and commercial organizations.

For more information, email AmeriSpeak-BD@norc.org or visit AmeriSpeak.norc.org.

If editors or reviewers are requesting anything more specific or any other detail, please reach out to us to make certain you are using accurate language.

NORC at the University of Chicago is best described as follows:

NORC at the University of Chicago is an independent research institution that delivers reliable data and rigorous analysis to guide critical programmatic, business, and policy decisions. Since 1941, NORC has conducted groundbreaking studies, created and applied innovative methods and tools, and advanced principles of scientific integrity and collaboration. Today, government, corporate, and nonprofit clients around the world partner with NORC to transform increasingly complex information into useful knowledge. Please visit www.norc.org for more information.

© 2022 Inwards-Breland DJ et al. *JAMA Network Open.*

APPENDIX

TECHNICAL OVERVIEW OF THE AMERISPEAK® PANEL NORC'S PROBABILITY-BASED HOUSEHOLD PANEL

Updated February 18, 2019
Prepared by J. Michael Dennis, Ph.D.

Funded and operated by NORC at the University of Chicago, AmeriSpeak® is a probability-based panel designed to be representative of the US household population. Randomly selected US households are sampled with a known, non-zero probability of selection from the NORC National Frame and address-based sample, and then contacted by US mail, telephone interviewers, overnight express mailers, and field interviewers (face to face). AmeriSpeak panelists participate in NORC studies or studies conducted by NORC on behalf of NORC's clients.

In 2018, the AmeriSpeak Panel expanded to approximately 30,000 households and will expand to 35,000 households in 2019. The AmeriSpeak Panel includes sample support for surveys of various segments through AmeriSpeak Latino, AmeriSpeak Teen, and AmeriSpeak Young Adult (which includes an oversample of African Americans, Hispanics, and Asians age 18-34). AmeriSpeak is also the probability-sample source for TrueNorth™, which combines probability-based AmeriSpeak and non-probability online samples using calibrating statistical weights derived from AmeriSpeak, the American Community Survey, and other data sources.[3]

Sample Frame

In order to provide a nationally representative sample, AmeriSpeak leverages the NORC National Frame, which provides sample coverage for over 97 percent of the U.S. households. The 2010 National Frame used a two-stage probability sample design to select a representative sample of households in the United States. The first stage—the sampling unit—is a National Frame Area (NFA), which is either an entire metropolitan area (made up of one or more counties) or a county (some counties were combined so that each NFA contains a population of at least 10,000). The largest NFAs with a population of at least 1,543,728 (0.5 percent of the 2010 Census U.S. population) were selected with certainty; these areas have a high-population density, and are dominated by tracts with street-style addresses. These areas contain 56 percent of the population within 8 percent of the geographic area of the United States. The remaining areas were stratified into areas where street-style addresses predominate, and the remaining areas, which are less likely to have street -style addresses. The latter stratum ("rural" areas) comprises 81 percent of the geographic area, but only 14 percent of the population.

Within the selected NFAs, the second stage sampling unit is a segment, defined in terms of either Census tracts or block groups, containing at least 300 housing units according to the 2010 Census. A stratified probability sample of 1,514 segments was selected with probability proportional to size. For most of the 1,514 segments, the U.S. Postal Service Delivery Sequence File (DSF) provided over 90 percent coverage of the segments in terms of city-style addresses that are geo-codeable. For the 123 segments where the DSF provided insufficient coverage, we enhanced the DSF address list with in-person listing. The National Frame contains almost 3 million households, including over 80,000 rural households added through the in-person listing.

---

[3] For more information, see "Estimation Methods for Nonprobability Samples with a Companion Probability Sample" authored by Michael Yang, N. Ganesh, Edward Mulrow, and Vicki Pineau. Published in the 2018 JSM Proceedings, Survey Research Methods Section. Alexandria, VA: American Statistical Association. 1715-1723. The paper is available at https://amerispeak.norc.org/research/. Please note that the TrueNorth™ trademarking was in progress when this paper was published. Hence, the authors do not reference the TrueNorth™ name on this paper.

© 2022 Inwards-Breland DJ et al. *JAMA Network Open*.

The National Frame involves addresses in almost every state. For the states that are not included in the National Frame, AmeriSpeak selected an address-based sample (ABS) in 2016 through 2018 from the USPS DSF to assure AmeriSpeak sample representation for all US States and Washington, DC.

In 2017, a targeted address-based sample was added to AmeriSpeak recruitment in order to develop a new Latino Panel with adequate representation of Spanish-language-dominant Hispanics. Census tracts with high incidence (at least 30%) of Spanish-dominant Hispanics were targeted for this recruitment. Furthermore, within these Census tracts, households that were flagged as Hispanic based on consumer vendor data (that are typically used for direct-mail marketing) were oversampled. This new AmeriSpeak Latino Panel contains approximately 5,400 Hispanic panelists with 24% of those panelists being Spanish-language dominant. As of February 2019, 11% of AmeriSpeak Panel (including the Latino Panel) recruited adults were sourced from the targeted address-based sample and 89% from the National Frame. Proper weights allow the full use of the combined sample.

Sample Selection for Panel Recruitment

The 2014-2018 AmeriSpeak Panel sample consists of nationally representative housing units drawn primarily from the 2010 NORC National Sample Frame. To create AmeriSpeak Latino and provide sample coverage for states where the National Frame is not operative, a secondary source is address-based sampling, which accounts for 12% of the 2014-2018 sample. The 2010 NORC National Sample Frame is stratified based on segment (Census tract or Census block group) characteristics such as age and race/ethnicity composition of the segment, and then, a stratified simple random sample of housing units is selected. Specifically, based on Census tract-level data, segments were classified as having a higher concentration of 18-24 year old adults or not, and a higher concentration of Hispanics, non-Hispanic African Americans, and other. Based on these strata definitions, 6 strata (2 based on age times 3 based on race/ethnicity) were used to oversample housing units in segments higher in young adults and/or Hispanics and non-Hispanic African-Americans. This is referred to as the initial sample or first stage of panel recruitment.

In the second stage of panel recruitment, initially sampled but nonresponding housing units are subsampled for a nonresponse follow-up (NRFU). At this stage, consumer vendor data are matched to housing units, and housing units that are flagged (based on consumer vendor data) as having a young adult (18-34 years of age) or minority (Hispanic and non-Hispanic African American) are oversampled for the NRFU. Overall, approximately one in five initially nonresponding housing units are subsampled for NRFU. Due to NRFU, these initially nonresponding housing units have a much higher selection probability compared to the housing units that were recruited during the first stage of panel recruitment. Note that a small fraction of initially nonresponding housing units are not eligible for NRFU due to these housing units being classified as "hard refusals" or having an appointment for a call back from NORC.

In summary, there are mainly two reasons why the sampling design for AmeriSpeak Panel recruitment deviates from Equal Probability of Selection Method (EPSEM) sampling: (a) oversampling of housing units in segments with a higher concentration of young adults and minorities results in the sample selection probabilities being higher for housing units in these segments; and (b) the nonresponse follow-up effort results in initially nonresponding housing units having a much higher selection probability. Furthermore, oversampling associated with NRFU results in higher selection probabilities for initially nonresponding housing units that are flagged (based on consumer vendor data) using demographics that are correlated with sample member's propensity to respond. The initial and NRFU sampling procedures are examined and modified each year to more efficiently recruit types of panelists who are less likely to respond based on their certain demographic characteristics.

AmeriSpeak Panel Recruitment Procedures

Recruitment is a two-stage process: initial recruitment using less expensive methods and then non-response follow-up using personal interviewers. For the initial recruitment, sample units are invited to join AmeriSpeak online by visiting the panel website AmeriSpeak.org or by telephone (in-bound/outbound

© 2022 Inwards-Breland DJ et al. *JAMA Network Open*.

supported). English and Spanish language are supported for both online and telephone recruitment. Study invitations are communicated via an over-sized pre-notification postcard, a USPS recruitment package in a 9"x12" envelope (containing a cover letter, a summary of the privacy policy, FAQs, and a study brochure), two follow-up post cards, and also contact by NORC's telephone research center for sample units matched to a telephone number.

The second-stage non-response follow-up targets a stratified random sub-sample of the non-responders from the initial recruitment. Units sampled for the non-response follow-up are sent by Federal Express a new recruitment package with an enhanced incentive offer. NORC field interviewers then make personal, face-to-face visits to the respondents' homes to encourage participation. NORC field interviewers administer the recruitment survey in-person using CAPI or else encourage the respondents to register at AmeriSpeak.org or call the toll-free AmeriSpeak telephone number to register.

Recruiting Non-Internet and "Net Averse" Households

Under certain conditions, AmeriSpeak gives panelists a choice regarding their preferred mode for future participation in AmeriSpeak surveys. As of February 2019, 83% of the active panelists were enrolled in AmeriSpeak to receive online surveys, while 17% of the active panelists agreed to participate in AmeriSpeak telephone mode surveys. For the 2016 through 2018 recruitment, respondents provided an option of online or telephone modes include: persons without internet access, persons whose only internet access is via a smartphone, and persons with internet access but unwilling to share an email address. A recruited household can consist of both web-mode and phone-mode panelists residing in the same household.

Impact of Non-Response Follow-up

The non-response follow-up (NRFU) is instrumental for producing a credible AAPOR response rate for the panel, boosting the panel recruitment response rate by a factor of 6.1 (AAPOR RR3, weighted to take into account selection probabilities). Additionally, NRFU reduces non-response bias significantly by improving the representativeness of the AmeriSpeak panel sample with respect to certain hard-to-reach segments of the population underrepresented by recruitment relying only mail and phone. NRFU improves representation for demographic segments (typically more reluctant to respond to surveys), including lower income households, cell-phone only households, renters, persons age 18 to 34, African Americans, Hispanics, and persons without a high school degree or have only a high school degree (no college). Even though NRFU panelists are more reluctant to complete surveys, the addition of NRFU panelists reduced total absolute bias on average 5-21% when compared to the initial stage recruits (among examined surveys).[4] Compared to panelists recruited in the initial stage, panelists recruited via the non-response follow-up campaign overall report more moderate opinions towards policy issues and are somewhat more conservative. Based on study specific findings, NRFU panelists report being less knowledgeable about science, report less interest in current events and topics in the news (such as climate change and energy resources), and are less likely to read a print newspaper (more likely to read the news online and use social media).[5] They are also more likely to attend church, less likely to be in favor of gun control policies, and more likely to eat at a fast food restaurant than the initial stage recruits. Accordingly, our extensive research on this topic illustrates that NRFU panelists make the substantive estimates in any AmeriSpeak study more inclusive and accurate.

AmeriSpeak Panel Recruitment Response Rate and Other Sample Metrics

---

[4] *See* "Nonresponse Follow-up Impact on AmeriSpeak Panel Sample Composition and Representativeness" authored by Ipek Bilgen, J. Michael Dennis, N. Ganesh. The paper is available at https://amerispeak.norc.org/research/;

Total Absolute Error = [Sum(|ACS 2016 Benchmark - Unweighted Variable Percentage Point|)]; see pages 8-13.

[5] *See* "The Undercounted: Measuring the Impact of 'Nonresponse Follow-up' on Research Data and Outcome Measures" authored by Ipek Bilgen, J. Michael Dennis, N. Ganesh. The paper will be soon available at https://amerispeak.norc.org/research/.

© 2022 Inwards-Breland DJ et al. *JAMA Network Open*.

The AAPOR RR3 (response rate) for the 2014-2018 panel recruitment 34.2% (weighted to take into account selection probabilities).[6] The estimated cumulative AAPOR RR3 for client surveys is 10% to 20% (varying according to study parameters and taking into account all sources of non-response including panel recruitment, panel household attrition, and survey participation).[7] NORC documented the AAPOR response rate calculation methodology for 2014-2015 recruitment.[8]

Key statistics with respect to the 2014-2018 recruited households are as follows: 52% recruited via the non-response follow-up recruitment using overnight Federal Express mailers and face-to-face methodology (with NORC field staff visiting households); 22% indicated a preference for the telephone mode of data collection for participating in AmeriSpeak studies; 25% of the recruited households are non-Internet[9]; 79% are cell-phone only or cell-phone mostly; 17% are African-American and 23% Hispanic; and 35% have household income below $30,000 (compared to CPS benchmark of 26%).[10]

Mixed-Mode Data Collection

Panelists may participate in two to three AmeriSpeak Panel studies per month via online (computer, tablet, or smartphones) or by CATI phone. CATI phone mode respondents represent a population currently under-represented in web panels that exclude non-internet households or "net averse" persons. NORC's telephone interviewers administer the phone mode of survey questionnaires using a data collection system supporting both the phone and web modes of data collection, providing an integrated sample management and data collection platform. For panelists using smartphones for web-mode AmeriSpeak surveys, the NORC survey system renders an optimized presentation of the survey questions for these mobile users. For general population client studies, approximately 17% of the completed interviews by the active panelists are completed via the telephone mode.

Panel Management Policies

NORC maintains strict rules to limit respondent burden and reduce the risk of panel fatigue. On average, AmeriSpeak panel members typically participate in AmeriSpeak web-based or phone-based studies two to three times a month.

Because the risk of panel attrition increases with the fielding of poorly constructed survey questionnaires, the AmeriSpeak team works with NORC clients to create surveys that provide an appropriate user experience for AmeriSpeak panelists. AmeriSpeak will not field surveys that in our professional opinion will result in a poor user experience for our panelists and in panel attrition.

---

[6] The response rate calculation incorporates the selection probabilities of the samples for the initial recruitment and non-response follow-up stages, as calculated by the US Bureau of the Census for the American Community Survey.

[7] A properly calculated cumulative AAPOR response rate for panel-based research takes into account all sources of non-response at each stage of the panel recruitment, management, and survey administration process. A common misapplication of the term "response rate" in online panel surveys is to represent the survey-specific cooperation rate as the "cumulative survey response rate."

[8] *See* "Response Rate Calculation Methodology for Recruitment of a Two-Phase Probability-Based Panel: The Case of AmeriSpeak" authored by Robert Montgomery, J. Michael Dennis, N. Ganesh. The paper is available at https://amerispeak.norc.org/research/.

[9] The non-internet households (HHs) are those that do not select "High-speed, broadband internet at home (such as cable or DSL)" or "Dial-up internet at home" response options when they are asked "What kind of internet access do you have? Please select all that apply" item in the recruitment survey. The non-internet HHs include those that only use internet on a cell connection or mobile phone.

[10] For transparency purposes, unweighted percentages are presented in this section. Hence, these results do not take into account oversampling and selection probabilities. The base weighted distributions that take take into account selection probabilities can be provided upon request.

© 2022 Inwards-Breland DJ et al. *JAMA Network Open.*

ABOUT NORC AT THE UNIVERSITY OF CHICAGO

As one of the world's foremost independent research institutions, NORC at the University of Chicago delivers objective data and meaningful analysis to help decision-makers and leading organizations make informed choices and identify new opportunities. Since 1941, NORC has applied sophisticated methods and tools, innovative and cost-effective solutions, and the highest standards of scientific integrity and quality to conduct and advance research on critical issues. Today, NORC expands on this tradition by partnering with government, business, and nonprofit clients to create deep insight across a broad range of topics and to disseminate useful knowledge throughout society.

Headquartered in downtown Chicago, NORC works in over 40 countries around the world, with additional offices on the University of Chicago campus, the DC metro area, Atlanta, Boston, and San Francisco.

ADDITIONAL RESOURCES

To learn more about AmeriSpeak or to share an RFP, please contact AmeriSpeak at AmeriSpeak-BD@norc.org. Information about AmeriSpeak capabilities and research papers are available online at AmeriSpeak.NORC.org.

© 2022 Inwards-Breland DJ et al. *JAMA Network Open.*
